# Diet and physical activity behaviors among women with polycystic ovary syndrome (PCOS): Are they different from healthy women?

**DOI:** 10.5935/1518-0557.20250058

**Published:** 2026

**Authors:** Saloni Kamboj, Taruna Arora, Dr Amlin, Anshu Jyoti, Gautam Ahuja, Spondita Banerjee, Rintu Kutum, Neena Malhotra

**Affiliations:** 1 Department of Obstetrics and Gynaecology, All India Institute Of Medical Sciences, New Delhi, India; 2 Reproductive Biology and Maternal Health, Child Health, Indian Council of Medical Research, New Delhi, India; 3 Department of Computer Science, Ashoka University, Haryana, India; 4 Koita Centre for Digital Health at Ashoka (KCDH-A) & Mphasis Lab for Applied AI & Tech Lab at Ashoka; 5 Trivedi School of Biosciences, Ashoka University

**Keywords:** PCOS women, diet, physical activity

## Abstract

**Objective:**

To investigate the diet and physical activity habits among women with polycystic ovary syndrome (PCOS) and compare them with age and BMI matched controls.

**Methods:**

This cross-sectional cohort study included 90 women with PCOS from a diverse population representing various socioeconomic backgrounds in both urban and rural areas. Ninety women with PCOS, diagnosed according to Rotterdam criteria, were matched for age and BMI with 270 healthy women from the same community who served as a reference group. Both groups completed interviews about their dietary habits and physical activity using a validated 126-item Food Frequency Questionnaire (FFQ) and Exercise questionnaire. Additionally, dietary assessment was performed using Diet-Cal Software.

**Results:**

Based on 24-hour dietary intake calculations, total calorie, carbohydrate, and fat consumption were higher, while fiber intake was lower in the PCOS group. When assessing and comparing the frequency of specific food groups using a food frequency questionnaire, certain foods-such as milk/milk products, processed foods, cereals, root/tubers, pulses, and nuts-were found to be more commonly consumed. Physical activity hours were significantly lower in the PCOS group. Univariate, multivariate, and explainable machine learning models were also developed to explore the relationship between dietary habits and physical activity in women with PCOS.

**Conclusions:**

The PCOS group differs from the control group in overall calorie, carbohydrate, fat, and fiber intake. The PCOS group also exhibited poorer physical activity behaviors compared to the control group.

## INTRODUCTION

Polycystic ovary syndrome (PCOS) is the most common endocrine disorder among women of reproductive age, involving reproductive, metabolic, and hormonal disturbances. It accounts for 70-80% of cases of anovulatory infertility (Altieri *et al.*, 2013; Lin *et al.*, 2019). The prevalence of PCOS varies depending on the defining criteria and ethnicity. It ranges from 5% to 10% according to NIH 1990 criteria; from 10% to 15% based on the AE-PCOS (Azziz *et al.*, 2006, 2009; Ün *et al.*, 2016) criteria; and from 6% to 21% with the ESHRE/ASRM (2003) (Rotterdam ESHRE/ASRM-Sponsored PCOS Consensus Workshop Group, 2004a, 2004b) criteria. Occurrence tends to increase with urbanization (Wang *et al.*, 2021). Importantly, PCOS affects not only women’s health but also has long-term consequences beyond reproductive age, including a higher incidence of type 2 diabetes mellitus, coronary heart disease, atherogenic dyslipidemia, cerebrovascular morbidity, and mental health issues such as anxiety and depression (Oliveira *et al.*, 2013; Lin *et al.*, 2021; Jurczewska *et al.*, 2023).

Currently, the exact cause of PCOS is not fully understood, but interactions between genetic and environmental factors are believed to play a role (Alomran & Estrella, 2023). These include family history, low birth weight, obesity, poor dietary habits, and low physical activity. About 50-60% of women with PCOS are overweight or obese. Obesity, especially visceral fat, contributes to metabolic and hormonal disturbances and reduces quality of life (Ehsani *et al.*, 2016), and it also leads to a poor response to pharmacological treatments for PCOS. Preliminary evidence indicates that women with PCOS are more prone to gaining weight (Kazemi *et al.*, 2022). However, there is controversy about whether dietary and physical activity behaviors influence the development of PCOS (Kazemi *et al.*, 2022). It remains unclear whether women with PCOS tend to eat poorer diets or engage in less physical activity, which might contribute to weight gain (Cutler *et al.*, 2019).

According to the American Society for Reproductive Medicine 2018 Guidelines, the first-line treatment for PCOS (Teede *et al.*, 2023) is lifestyle modification, including diet management and exercise, with weight control being especially crucial for PCOS patients because it improves metabolic, hormonal, and reproductive parameters, as well as quality of life. Various studies examining different diet compositions-such as altering the amount or type of dietary protein, fat, or carbohydrate, including high-protein diets, low-glycemic index (GI) diets, very-low-carbohydrate diets, or diets that modify the fatty acid profile-have not identified a specific optimal dietary composition.

Despite many recommendations, there is a notable lack of literature on the distribution of macronutrients, micronutrients, and fiber, as well as physical activity targets, for women with PCOS, which poses a significant challenge for researchers and clinicians. Therefore, we evaluated dietary habits and physical activity levels among women with PCOS and compared them to those of controls from the same community.

## MATERIAL AND METHODS

This prospective cohort study included women diagnosed with PCOS aged between 18-40 years who fulfilled the Rotterdam criteria. It was a sub-study from a nationwide task force study funded by the ICMR. Participants were recruited from both rural and urban sectors of Delhi-NCR after providing written informed consent. Controls were women without PCOS recruited from the same communities as the study subjects to match in terms of socio-economic features. The study was conducted over 18 months. Women with untreated thyroid dysfunction, diabetes mellitus, hyperprolactinemia, premature ovarian failure, or those taking anti-obesity or insulin-sensitizing drugs in the past three months, as well as pregnant or lactating women, were excluded. The study commenced after ethics approval. Both groups were surveyed about their dietary patterns and physical activity behaviors using a validated 126-item Food Frequency Questionnaire and Exercise questionnaire. Seasonal variations were considered when designing these questionnaires. Questions were administered through home visits via call recall method.

The dietary and physical activity patterns of women with PCOS were compared to those of controls. Findings were correlated with clinical and biochemical parameters, including anthropometry and metabolic indices such as fasting and postprandial glucose, insulin levels, and lipid profile.

### Statistics

Categorical variables were presented as numbers and percentages (%), while quantitative data with non-normal distribution were expressed as medians with 25th and 75th percentiles (interquartile range). Data normality was assessed using the Shapiro-Wilk test. For data that was not normally distributed, non-parametric tests were used. Comparison of quantitative variables that were not normally distributed was performed using the Mann-Whitney test. Qualitative variables were compared using the Chi-Square test, with Fisher’s exact test applied if any cell had an expected frequency of less than 5. Spearman’s rank correlation coefficient was used to examine the relationship between dietary habits, physical activity, clinical parameters, and lipid profile. Data analysis was conducted using the Statistical Package for Social Sciences (SPSS) software (IBM, Chicago, USA, version 25.0). A *p*-value of less than 0.05 was considered statistically significant. Additionally, multivariate and explainable machine learning models were developed to explore the relationship between dietary habits and physical activity in women with PCOS.

## RESULTS

When the frequency of food habits was compared between women with PCOS and controls, a notable difference was observed in the frequency of milk and milk products, processed foods, green leafy vegetables, roots, and tubers (Table 1). According to 24-hour dietary intake calculations, total calorie intake, carbohydrate intake (g/d), fat intake (g/d), and added sugars were higher in the PCOS group (Table 1).

**Table 1:** Comparison of dietary habits between women with PCOS and healthy controls.

Dietary habits	PCOS (n=90)	Healthy Controls (n=270)	Total	p value
**Frequency of dairy product intake**				
Daily	87 (96.67%)	41 (15.19%)	128 (35.56%)	<0.0001^†^
2-3 times in a week	3 (3.33%)	158 (58.52%)	161 (44.72%)	
Once weekly	0 (0%)	71 (26.30%)	71 (19.72%)	
**Frequency of processed food intake**				
Daily	74 (82.22%)	0 (0%)	74 (20.56%)	<0.0001^*^
2-3 times in a week	6 (6.67%)	0 (0%)	6 (1.67%)	
Once weekly	10 (11.11%)	2 (0.74%)	12 (3.33%)	
2-3 times in a month	0 (0%)	55 (20.37%)	55 (15.28%)	
Once monthly	0 (0%)	132 (48.89%)	132 (36.67%)	
2-3 times in year	0 (0%)	81 (30%)	81 (22.50%)	
**Frequency of cereal intake**				
Daily	90 (100%)	270 (100%)	360 (100%)	----
**Frequency of green leafy vegetable intake**				
Daily	8 (8.89%)	201 (74.44%)	209 (58.06%)	<0.0001^†^
2-3 times in a week	37 (41.11%)	67 (24.81%)	104 (28.89%)	
Once weekly	45 (50%)	2 (0.74%)	47 (13.06%)	
**Frequency of root and tuber intake**				
Daily	25 (27.78%)	0 (0%)	25 (6.94%)	<0.0001^*^
2-3 times in a week	60 (66.67%)	0 (0%)	60 (16.67%)	
Once weekly	5 (5.56%)	2 (0.74%)	7 (1.94%)	
2-3 times in month	0 (0%)	59 (21.85%)	59 (16.39%)	
Once monthly	0 (0%)	128 (47.41%)	128 (35.56%)	
2-3 times in year	0 (0%)	81 (30%)	81 (22.50%)	
**Frequency of fruit intake**				
*Daily*	0 (0%)	135 (50%)	135 (37.50%)	<0.0001^†^
*2-3 times in a week*	2 (2.22%)	77 (28.52%)	79 (21.94%)	
*Once weekly*	13 (14.44%)	52 (19.26%)	65 (18.06%)	
*2-3 times in month*	75 (83.33%)	6 (2.22%)	81 (22.50%)	
**Frequency of pulses**				
*Daily*	33 (36.67%)	170 (62.96%)	203 (56.39%)	<.0001^†^
*2-3 times in a week*	56 (62.22%)	76 (28.15%)	132 (36.67%)	
*Once weekly*	1 (1.11%)	24 (8.89%)	25 (6.94%)	
**Frequency of nut intake**				
*Once weekly*	2 (2.22%)	85 (31.48%)	87 (24.17%)	<.0001^^*^^
*2-3 times in month*	5 (5.56%)	185 (68.52%)	190 (52.78%)	
*Once monthly*	81 (90%)	0 (0%)	81 (22.50%)	
*2-3 times in year*	2 (2.22%)	0 (0%)	2 (0.56%)	
**Calorie intake (Kilocalorie per day)**	1940 (1812-2115.5)	1666 (1584-1737.75)	1693 (1602-1832)	<.0001^‡^
**Fat intake (gm/day)**	80 (74.25-90)	74 (66-84)	77 (68-85)	<.0001^‡^
**Protein intake (gm/day)**	55 (50-57)	51 (48-56)	52 (48-57)	0.002^‡^
**Carbohydrate intake (gm/day)**	246.5 (227.75-278)	194 (179.25-211)	203.5 (183.75-222)	<.0001^‡^
**Fiber intake (gm/day)**	4 (2.25-6)	7 (6-9)	7 (5-8)	<.0001^‡^
**Added sugar**	54 (49.25-58)	39 (33.25-44)	42.5 (36-48.25)	<.0001^‡^

‡ Mann Whitney test,

* Fisher's exact test,

† Chi square test.

Physical activity hours were significantly lower in the PCOS group. Parameters compared included type of workout, preferred mode of commuting to work, physical activity hours, screen time, and sitting time (Table 2).

**Table 2:** Comparison of physical activity levels between women with PCOS and healthy controls.

Physical activity levels	PCOS (n=90)	Healthy Controls (n=270)	Total	p value
**Type of workout**				
*No exercise*	40 (44.44%)	50 (18.52%)	90 (25%)	<.0001^*^
*Walking*	32 (35.56%)	94 (34.81%)	126 (35%)	
*Swimming*	0 (0%)	1 (0.37%)	1 (0.28%)	
*Cycling*	2 (2.22%)	16 (5.93%)	18 (5%)	
*Gym*	8 (8.89%)	73 (27.04%)	81 (22.50%)	
*Others*	8 (8.89%)	36 (13.33%)	44 (12.22%)	
**Preferred mode**				
*Car*	69 (76.67%)	67 (24.81%)	136 (37.78%)	<.0001^†^
*Walking*	21 (23.33%)	203 (75.19%)	224 (62.22%)	
**Physical activity (hours/day)**	0 (0-0.5)	0.75 (0-1)	0.5 (0-1)	<.0001^‡^
**Sitting time (hours)**	17 (16-18)	16 (15-18)	17 (15-18)	0.111^‡^
**Sleeping time (hours)**	7 (6-8)	7 (6-8)	7 (6-8)	0.31^‡^
**Screen time (hours)**	7 (6-8)	1 (0-1)	1 (0-2.25)	<.0001^‡^

‡ Mann Whitney test,

* Fisher's exact test,

† Chi-square test.

Anthropometric variables such as BMI, waist circumference, and WHR, along with clinical variables like SBP and DBP, were higher in cases than in controls (Table 3). When food and physical activity behaviors were linked with anthropometric and biochemical lab parameters, there was a statistically significant correlation between fasting insulin levels and calorie intake, fat intake, protein intake, and added sugar intake. Additionally, diastolic blood pressure levels were associated with carbohydrate intake (Table 4).

**Table 3:** Anthropometric parameters in women with PCOS and healthy controls.

Variable	PCOS	Healthy Controls	p value
Age (years)	27.17±6.30	27.90±6.18	0.3064
BMI (Kg/m^2^)	25.64±4.34	24.44±4.18	**0.041**
Waist circumference (cm)	81.42±11.70	78.98±12.06	**0.041**
Waist-to-Height ratio	0.39±0.06	0.32±0.06	**0.013**
Systolic blood pressure (mmHg)	116.94±12.15	115.86±11.80	**0.0441**
Diastolic blood pressure (mmHg)	76.07±8.51	75.10±7.71	**0.0077**

**Table 4:** Relationship between dietary habits, physical activity, and clinical parameters.

Variables	Body mass index (kg/m^2^)	Waist circumference (cm)	Systolic blood pressure (mmHg)	Diastolic blood pressure (mmHg)	Fasting blood sugar (mg/dL)	Postprandial blood sugar (mg/dL)	Fasting insulin (mIU/L)	Insulin PP 120 (mIU/L)
**Calorie intake (Kilocalorie per day)**								
*Correlation coefficient*	0.023	-0.074	0.012	-0.111	0.006	-0.009	0.199	0.163
*p-value*	0.826	0.488	0.909	0.296	0.954	0.930	0.061	0.128
**Fat intake (gm/day)**								
*Correlation coefficient*	-0.110	-0.145	0.050	0.025	0.054	-0.062	0.222	0.218
*p-value*	0.300	0.174	0.636	0.811	0.615	0.562	0.037	0.040
**Protein intake (gm/day)**								
*Correlation coefficient*	-0.018	-0.067	-0.109	0.019	0.161	0.148	0.246	0.139
*p-value*	0.867	0.531	0.306	0.861	0.128	0.164	0.021	0.194
**Carbohydrate intake (gm/day)**								
Correlation coefficient	0.048	-0.084	-0.020	0.237	-0.067	-0.044	0.109	0.091
p-value	0.655	0.430	0.854	0.024	0.529	0.682	0.311	0.396
**Fibre intake (gm/day)**								
*Correlation coefficient*	0.058	-0.033	0.095	-0.174	-0.027	-0.171	-0.052	-0.024
*p-value*	0.585	0.755	0.373	0.101	0.799	0.106	0.629	0.821
**Added sugar**								
*Correlation coefficient*	-0.002	-0.004	-0.082	-0.095	0.148	0.126	0.376	0.261
*p-value*	0.988	0.967	0.442	0.373	0.165	0.238	0.0003	0.014
**Physical activity (hours/day)**								
*Correlation coefficient*	0.063	0.012	0.171	0.133	0.078	0.072	-0.042	-0.116
*p-value*	0.557	0.909	0.106	0.211	0.464	0.499	0.695	0.277
*Sitting time (hours)*								
*Correlation coefficient*	0.162	0.044	0.010	0.045	-0.188	-0.103	0.033	0.010
*p-value*	0.126	0.679	0.928	0.670	0.076	0.332	0.760	0.925
**Sleeping time (hours)**								
*Correlation coefficient*	-0.050	-0.061	0.101	0.007	0.088	0.048	0.094	0.183
*p-value*	0.640	0.567	0.344	0.949	0.410	0.651	0.379	0.087
**Screen time (hours)**								
*Correlation coefficient*	0.029	0.001	0.161	0.018	-0.132	-0.020	0.111	0.037
*p-value*	0.788	0.995	0.129	0.865	0.216	0.849	0.301	0.727

Spearman’s rank correlation coefficient.

When the lipid profile was correlated with food habits and physical activity levels, hours of physical activity were significantly negatively correlated with total cholesterol levels. (Table 5).

**Table 5:** Relationship between dietary habits, physical activity, and lipid profile.

Variables	Cholesterol (mg/dL)	HDL (mg/dL)	Triglycerides (mg/dL)	LDL (mg/dL)
**Calorie intake (Kilocalorie per day)**				
*Correlation coefficient*	-0.026	0.032	-0.064	-0.060
*p-value*	0.810	0.767	0.546	0.573
**Fat intake (gm/day)**				
*Correlation coefficient*	0.058	-0.038	-0.039	0.088
*p-value*	0.584	0.720	0.713	0.411
**Protein intake (gm/day)**				
*Correlation coefficient*	0.017	-0.136	0.132	0.026
*p-value*	0.871	0.202	0.216	0.805
**Carbohydrate intake (gm/day)**				
*Correlation coefficient*	-0.101	0.039	-0.147	-0.121
*p-value*	0.345	0.713	0.167	0.254
**Fiber intake (gm/day)**				
*Correlation coefficient*	0.042	-0.094	-0.009	0.096
*p-value*	0.692	0.376	0.930	0.365
**Added sugar**				
*Correlation coefficient*	0.077	0.007	-0.172	0.131
*p-value*	0.471	0.947	0.105	0.217
**Physical activity (hours/day)**				
*Correlation coefficient*	-0.201	-0.087	-0.055	-0.163
*p-value*	0.058	0.414	0.607	0.124
**Sitting time (hours)**				
*Correlation coefficient*	-0.089	-0.101	-0.036	0.004
*p-value*	0.406	0.342	0.735	0.972
**Sleeping time (hours)**				
*Correlation coefficient*	0.026	-0.040	-0.100	0.066
*p-value*	0.811	0.710	0.347	0.537
**Screen time (hours)**				
*Correlation coefficient*	-0.024	-0.052	-0.076	-0.009
*p-value*	0.819	0.624	0.479	0.930

Spearman’s rank correlation coefficient.

### Machine learning-based multivariate analysis of food habits, physical activity, and lab parameters

**Figure 1:**
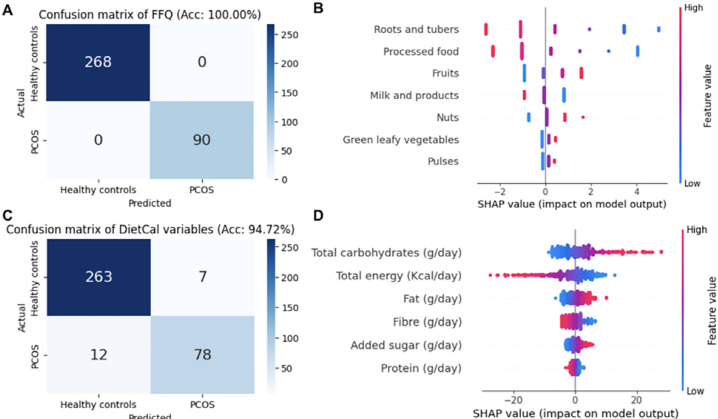
Multivariate logistic regression model and SHAP analysis of the model: A. Confusion matrix showing logistic models based on food frequency questionnaire parameters (features). B. Summary dot plot of the SHAP values for the FFQ-based logistic model, illustrating feature impact (top variables are the most influential to the model’s performance). C. Confusion matrix based on the DietCal variables estimated from the FFQ variables. D. Summary dot plot of the SHAP values for the DietCal based on the multivariate model.

Multivariate logistic models and eXplainable AI algorithms such as SHAP (SHapley Additive exPlanations) were used to delineate the contribution of the parameters of food habits (Food Frequency Questionnaire - FFQ, and DietCal variables). The FFQ variables-based logistic models show an accuracy of 100% (Figure 1A), and SHAP analysis of the model shows the frequency of roots and tubers, processed foods, fruits, and milk and dairy products as the top contributors to the model (Figure 1B). On the other hand, the DietCal variables-based model shows an accuracy of 94.72% (Figure 1C), and SHAP analysis highlights the total carbohydrates (g/day) and total energy (kcal/day) as the two most influential variables contributing to the model’s performance.

**Figure 2:**
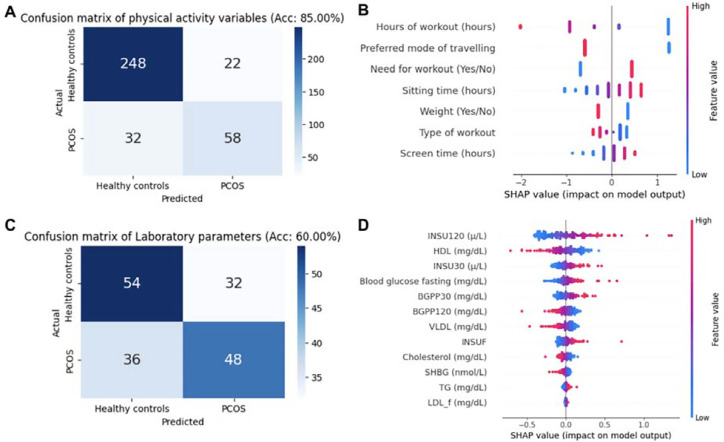
Multivariate logistic regression model and SHAP analysis of the model: A. Confusion matrix showing logistic models based on physical activity features. B. Summary dot plot of the SHAP values of the physical activity-based logistic model, illustrating feature impact (top variables are the most impactful). C. Confusion matrix based on the multivariate logistic model using laboratory parameters. D. Summary dot plot of the SHAP values for laboratory features in the multivariate model.

The multivariate model based on physical activity and laboratory parameters showed an overall accuracy of 85% (5-Fold CV accuracy) and 60% (5-Fold CV accuracy), respectively (Figures 2A and 2C). Overall, the hours of workout (hours), preferred mode of travel, and need for workout (yes/no) are the top three features based on the SHAP value analysis (Figure 2B). In the case of laboratory parameter-based multivariate logistic regression analysis, INSU120, HDL, and INSU30 are the top variables impacting the model based on the SHAP analysis (Figure 2D).

## DISCUSSION

PCOS is a lifestyle disorder characterized by a vicious cycle of altered eating and physical activity habits that contribute to weight gain and abnormal metabolic parameters, especially in obese individuals with the condition. Recognizing these patterns can help manage not only weight loss but also improve hormonal imbalance and reproductive health.

Our comparative data show differences between women with PCOS and those without based on two key lifestyle factors: physical activity and dietary intake. This study was conducted in a well-defined cohort that includes both rural and urban populations within a specific geographic area. The study population and controls had similar socio-economic status; therefore, dietary intake was consistent across various food groups. Our participants were young adults, with an average age of 29.02 years for women with PCOS and 31.32 years for healthy controls, which helps minimize age-related differences.

Our observations regarding anthropometric indices in women with and without PCOS align with other studies (Altieri *et al.*, 2013; Lin *et al.*, 2019; Wang *et al.*, 2021). Current findings, along with previous systematic reviews and meta-analyses, show similar links between poor diet and/or lack of exercise in the PCOS population (Lin *et al.*, 2021). Based on 24-hour dietary recall, energy intake was compared between the two groups. There is evidence of higher daily calorie intake in the PCOS group and lower energy consumption in healthy women. Increased calorie intake contributes to an increase in BMI, which matches our finding of higher BMI in the PCOS population. This supports previous studies where PCOS participants reported more weight gain, reflected by increased abdominal obesity measured through waist circumference (Jurczewska *et al.*, 2023).

Similar results aligning with previous studies showed a significant increase in carbohydrate intake among the PCOS population (PCOS: 251.46±39.11, Controls: 195.24±17.94, *p*<0.0001). Newer research indicates that a diet high in refined carbohydrates contributes to obesity, abnormal adipokine release, insulin resistance, neuroinflammation, and related diseases in both acute and chronic models (Oliveira *et al.*, 2013). Furthermore, our observations show that intake of simple carbohydrates exceeds that of complex ones when assessed via FFQ. Based on frequency, the types of food consumed by the population demonstrated a higher correlation with processed foods, cereals, roots and tubers, and added sugars (*p*<0.0001) in women with PCOS compared to those without.

Additionally, while assessing fat consumption, both methods-FFQ and 24-Hour dietary recall (PCOS: 82.4±10.47, controls: 74.97±9.66, *p*<0.0001)-showed that women with PCOS reportedly had increased intake of fat and refined cooking oils. Women with PCOS also have lower overall diet quality and poorer dietary intakes, such as higher cholesterol consumption, compared to those without PCOS (Alomran & Estrella, 2023). A study by Ehsani et al. revealed that a diet high in fried vegetables, vegetable oils (except olive oil), salty snacks, legumes, eggs, fast foods, onion, and garlic-and low in sweets, highor low-fat dairy products, cruciferous vegetables, simple sugars, and honey-was positively associated with the visceral adiposity index, another marker of abdominal obesity in women with PCOS (Ehsani *et al.*, 2016). Our population also exhibited similar patterns, with higher consumption frequencies of certain food groups, such as dairy products and refined oils. Hypercholesterolemia, another common complaint among women with PCOS, has been linked to the development of cardiometabolic and reproductive disruptions, type 2 diabetes, and hyperandrogenemia in PCOS (Kazemi *et al.*, 2022).

We observed that our participants had lower dietary fiber intake (24g/day) than the controls, aligning with previous findings in Canadian women reported by Cutler *et al.* (2019). Even Indian women with PCOS did not meet the dietary fiber recommendations in this study. Overall, our findings emphasize that women with PCOS consume less fiber, which increases their risk for insulin resistance, altered blood glucose levels, higher serum androgen levels, and systemic inflammation. All these factors are directly linked to the development of PCOS (Parker *et al.*, 2022) Therefore, women with PCOS are at greater risk for adverse cardiovascular profiles compared to women without PCOS (Sabag *et al.*, 2024).

We observed differences in protein intake between women with and without PCOS, unlike another study, with higher protein intake in the PCOS group (Kazemi *et al.*, 2022). Protein and amino acid consumption are known to enhance insulin secretion, which relates to a compensatory increase in insulin clearance, leading to lower plasma insulin levels (Nesti *et al.*, 2019; Tricò *et al.*, 2019). Furthermore, consuming protein-rich diets correlates with a reduction in carbohydrate intake, which could improve insulin sensitivity, boost pancreatic β-cell function, and increase endogenous insulin clearance (Brinkworth *et al.*, 2004). Protein-rich diets are linked to significantly more favorable insulin and HOMA-IR levels, although they have similar effects on body weight, abdominal obesity, lipid metabolism, and sex hormone levels (Wang *et al.*, 2024).

Our study showed that exercise and workout routines differ significantly between PCOS and non-PCOS groups. Overall activities, such as the need for exercise (*p*<0.0001), leisure time, and commuting to moderate-to-vigorous physical activities, including types of workouts (*p*<0.0001) and preferred modes of commute to work (*p*<0.0001), were much lower in PCOS cases. Physically active hours (hours per day) (0.21) were significantly lower in women with PCOS compared to the normal group (0.61). This aligns with other research reporting differences in sedentary and physical activity patterns in women with PCOS, which have been linked to an increased risk of morbidity from various causes (Eleftheriadou *et al.*, 2012; Chau *et al.*, 2013; Lin *et al.*, 2021).

Our multivariate logistic regression analysis using explainable machine learning methods like SHAP (SHapley Additive exPlanations) highlights food habit parameters based on FFQ that together have a discriminatory effect between PCOS and healthy controls. Additionally, we found that FFQ parameters have a better classification ability than the DietCal parameters. Parameters related to physical activity, such as hours of workout, preferred mode of travel, and need for exercise, were identified as contributing factors in distinguishing PCOS from healthy controls.

The strengths of our study included the sample size, a well-defined population of women with and without PCOS, and the prospective data collection. We explored differences in dietary intake across various food groups, eating behaviors, physical activities, and quality of life between women with PCOS and those without, and correlated these with metabolic parameters. Our findings support recent evidence-based guidelines advocating for the adoption and maintenance of healthy lifestyle habits among women with PCOS, following national recommendations for healthy lifestyle practices.

However, some limitations included a preferred larger sample size and the use of self-reported questionnaires, which might have led to over-reporting healthy behaviors and underreporting unhealthy behaviors. The FFQ only examined certain types of food products, so we were unable to analyze differences in nutrients such as minerals, vitamins, or micronutrients, which limited the comparisons of our findings.

Future studies on how changes in diet and physical activity will improve outcomes across different PCOS phenotypes are needed, especially given the recent international evidence-based guidelines for PCOS (Teede *et al.*, 2023).

## CONCLUSION

Our findings expand on previous research that highlights notable differences in dietary and physical activity behaviors between women with and without PCOS. They emphasize the practicality of applying healthy lifestyle recommendations from the recent International Evidence-based Guideline for the Assessment and Management of PCOS. Additionally, these observations highlight the vital role of nutrition professionals in providing evidence-based care to women with PCOS, supporting them in reaching healthy lifestyle goals and making informed choices to enhance both their shortand long-term reproductive and metabolic health.
